# Supplementary material for: The influence of muscle mass on the coordination required for efficient movement
Source: Front Physiol. 2026 Sep 11;17:1879888. doi: 10.3389/fphys.2026.1879888 (PMC13612335; doi:10.3389/fphys.2026.1879888)
Supplement: Supplementary file 1 [file DataSheet1.pdf]

The influence of muscle mass on the coordination required for efficient movement

Table S1. Characteristics of the 12 participants.

Label	Gender	Age (years)	Body mass (kg)	Height (m)
S02	male	28	68.0	1.67
S03	female	30	65.5	1.67
S04	male	33	68.0	1.75
S05	female	43	64.4	1.74
S06	male	32	63.0	1.83
S07	male	37	71.8	1.73
S11	male	31	82.8	1.83
S12	female	24	65.8	1.71
S13	female	32	63.5	1.68
S14	male	24	78.9	1.78
S15	female	26	58.0	1.67
S17	female	26	80.7	1.73

Table S2. Discretization-sensitivity analysis for the muscle-mass model. Differences in muscle activation were calculated relative to the $n_{\text{seg}} = 10$ solution. Muscle activation is dimensionless.

Muscle	RMS difference		Maximum absolute difference	
	3 vs. 10	5 vs. 10	3 vs. 10	5 vs. 10
Tibialis anterior	8.58×10^{-10}	5.74×10^{-10}	4.61×10^{-9}	3.08×10^{-9}
Medial gastrocnemius	2.52×10^{-9}	1.70×10^{-9}	6.31×10^{-9}	4.16×10^{-9}
Lateral gastrocnemius	1.73×10^{-9}	1.16×10^{-9}	1.06×10^{-8}	6.75×10^{-9}
Soleus	2.61×10^{-9}	6.02×10^{-10}	2.36×10^{-8}	4.17×10^{-9}
Vastus medialis	2.31×10^{-6}	6.82×10^{-9}	2.05×10^{-5}	4.62×10^{-8}
Rectus femoris	2.69×10^{-6}	8.13×10^{-9}	2.66×10^{-5}	7.60×10^{-8}
Vastus lateralis	2.21×10^{-6}	1.37×10^{-8}	1.96×10^{-5}	6.57×10^{-8}
Biceps femoris long head	1.68×10^{-6}	4.29×10^{-9}	1.59×10^{-5}	2.90×10^{-8}
Semitendinosus	1.03×10^{-7}	5.21×10^{-9}	1.01×10^{-6}	2.00×10^{-8}
Gluteus maximus	2.01×10^{-6}	5.89×10^{-9}	1.80×10^{-5}	4.46×10^{-8}
Mean across selected muscles	1.10×10^{-6}	4.81×10^{-9}	1.02×10^{-5}	3.00×10^{-8}

Table S3. Statistical results for the effects of muscle-mass scale and cycling condition on PC loading scores across PCs 1–25. For all PCs, the mass effect was tested with $F(4, 687)$ and the cycling-condition effect was tested with $F(3, 687)$. Raw p-values and Benjamini–Hochberg false-discovery-rate (BH-FDR) corrected p-values are reported. Effect sizes are reported as partial eta squared (η_p^2) and Cohen's f.

	Muscle-mass scale						Cycling condition					
PC	F	raw p	FDR p	η_p^2	f	Sig.	F	raw p	FDR p	η_p^2	f	Sig.
1	0.25	0.909	0.993	0.001	0.038	No	350.50	5.10×10^{-138}	1.27×10^{-136}	0.605	1.237	Yes
2	20.58	4.94×10^{-16}	2.06×10^{-15}	0.107	0.346	Yes	71.96	1.78×10^{-40}	2.22×10^{-39}	0.239	0.561	Yes
3	0.69	0.597	0.932	0.004	0.064	No	15.19	1.40×10^{-9}	2.69×10^{-9}	0.062	0.258	Yes
4	49.09	2.43×10^{-36}	2.02×10^{-35}	0.222	0.535	Yes	32.63	9.96×10^{-20}	4.15×10^{-19}	0.125	0.378	Yes
5	79.82	1.26×10^{-55}	1.57×10^{-54}	0.317	0.682	Yes	9.94	2.02×10^{-6}	2.97×10^{-6}	0.042	0.208	Yes
6	139.22	4.25×10^{-87}	1.06×10^{-85}	0.448	0.900	Yes	40.79	2.88×10^{-24}	1.44×10^{-23}	0.151	0.422	Yes
7	11.61	3.99×10^{-9}	1.25×10^{-8}	0.063	0.260	Yes	44.76	1.99×10^{-26}	1.24×10^{-25}	0.163	0.442	Yes
8	29.45	1.26×10^{-22}	7.87×10^{-22}	0.146	0.414	Yes	11.76	1.60×10^{-7}	2.67×10^{-7}	0.049	0.227	Yes
9	14.78	1.36×10^{-11}	4.85×10^{-11}	0.079	0.293	Yes	59.65	2.80×10^{-34}	2.33×10^{-33}	0.207	0.510	Yes
10	4.87	7.11×10^{-4}	0.002	0.028	0.168	Yes	11.08	4.12×10^{-7}	6.44×10^{-7}	0.046	0.220	Yes
11	5.70	1.62×10^{-4}	4.49×10^{-4}	0.032	0.182	Yes	7.90	3.47×10^{-5}	4.20×10^{-5}	0.033	0.186	Yes
12	21.65	7.70×10^{-17}	3.85×10^{-16}	0.112	0.355	Yes	13.55	1.34×10^{-8}	2.40×10^{-8}	0.056	0.243	Yes
13	0.78	0.541	0.932	0.004	0.067	No	18.43	1.64×10^{-11}	3.74×10^{-11}	0.074	0.284	Yes
14	4.92	6.50×10^{-4}	0.002	0.028	0.169	Yes	22.02	1.27×10^{-13}	3.96×10^{-13}	0.088	0.310	Yes
15	0.50	0.732	0.993	0.003	0.054	No	8.94	8.09×10^{-6}	1.12×10^{-5}	0.038	0.198	Yes
16	0.06	0.993	0.993	0.000	0.019	No	15.62	7.76×10^{-10}	1.62×10^{-9}	0.064	0.261	Yes
17	0.84	0.499	0.932	0.005	0.070	No	19.42	4.30×10^{-12}	1.08×10^{-11}	0.078	0.291	Yes
18	0.74	0.567	0.932	0.004	0.065	No	31.30	5.67×10^{-19}	2.02×10^{-18}	0.120	0.370	Yes
19	2.19	0.069	0.143	0.013	0.113	No	21.92	1.44×10^{-13}	4.01×10^{-13}	0.087	0.309	Yes
20	0.13	0.970	0.993	0.001	0.028	No	7.07	1.10×10^{-4}	1.26×10^{-4}	0.030	0.176	Yes
21	0.17	0.953	0.993	0.001	0.032	No	4.50	0.004	0.004	0.019	0.140	Yes
22	0.42	0.798	0.993	0.002	0.049	No	6.14	4.03×10^{-4}	4.39×10^{-4}	0.026	0.164	Yes
23	0.31	0.870	0.993	0.002	0.043	No	7.89	3.53×10^{-5}	4.20×10^{-5}	0.033	0.186	Yes
24	0.22	0.930	0.993	0.001	0.035	No	8.50	1.51×10^{-5}	1.99×10^{-5}	0.036	0.193	Yes
25	0.07	0.992	0.993	0.000	0.020	No	2.41	0.066	0.066	0.010	0.102	No

Note. Sig. indicates statistical significance after Benjamini–Hochberg FDR correction at $\alpha = 0.05$. For the muscle-mass scale effect, PCs 2, 4–12, and 14 remained significant after FDR correction. For the cycling-condition effect, PCs 1–24 remained significant after FDR correction.

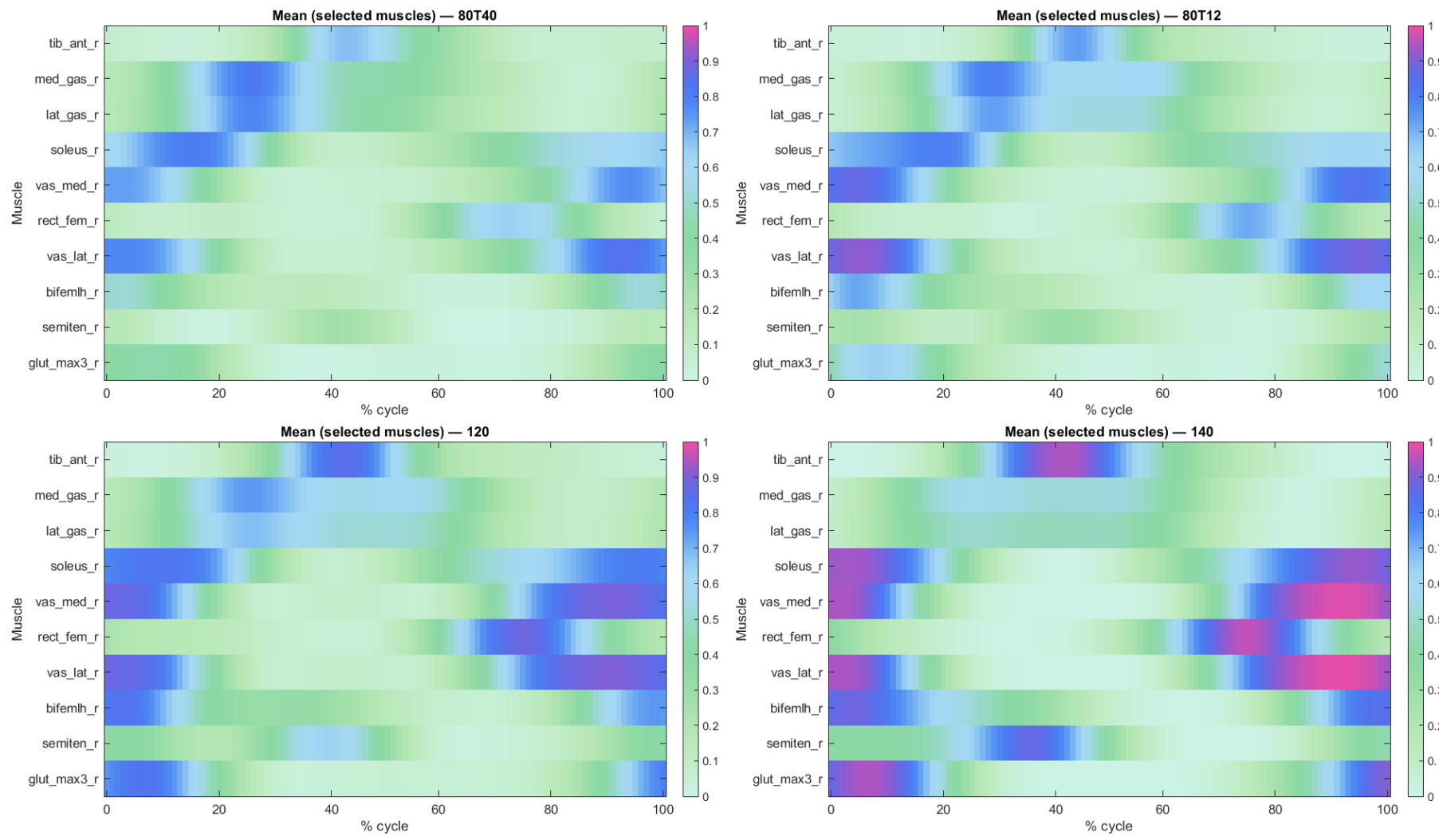

Fig. S1. The means of predicted muscles activations for all participants for selected muscles for the four tested cycling conditions. 3 conditions were for cycling against a crank torque of 12 N m, and at cadences of 80, 120 and 140 r.p.m. (referred to as 80T12, 120 and 140, respectively), and one condition was at a cadence of 80 r.p.m. and crank torque of 40 N m, referred to as 80T40.

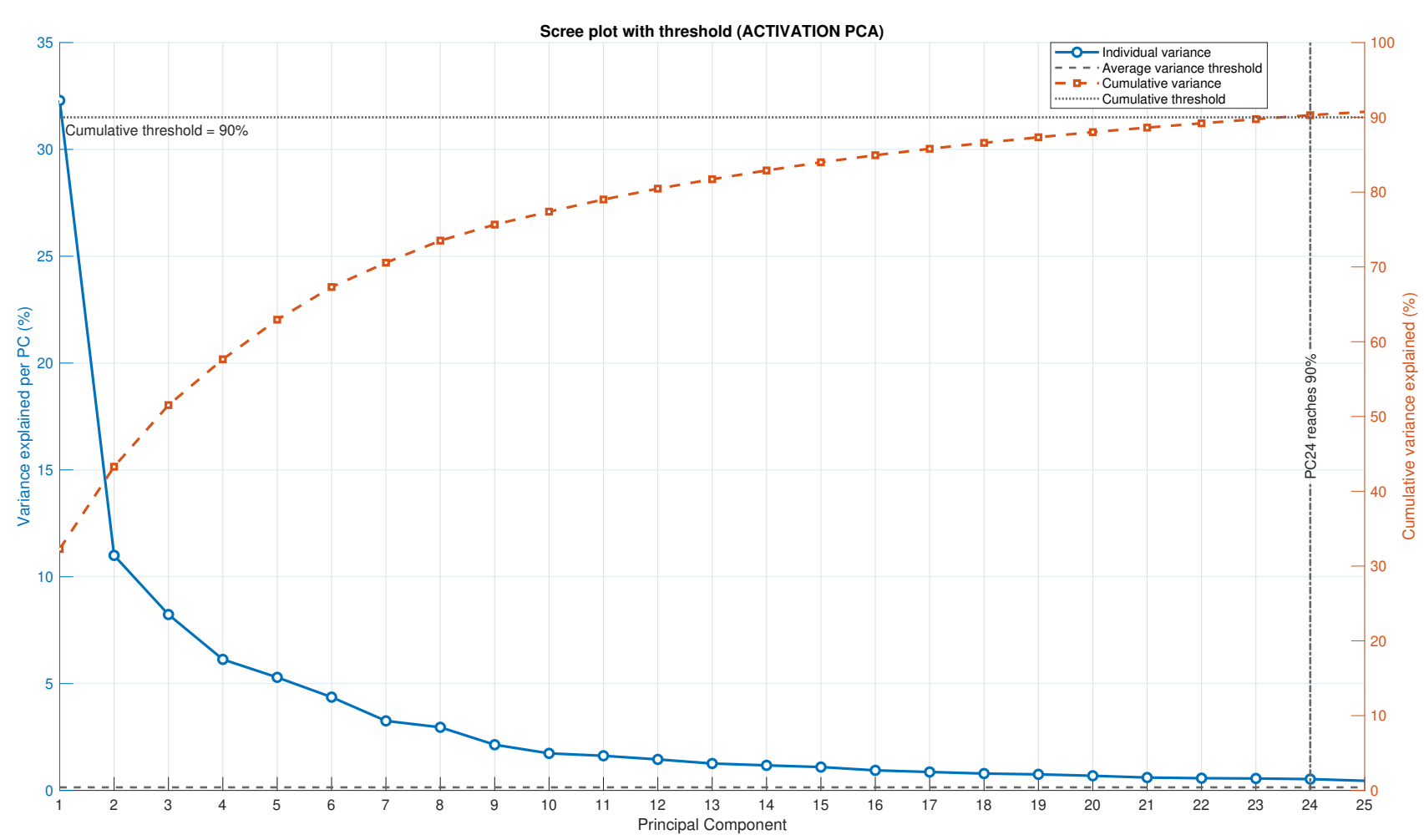

Fig. S2. Scree plot showing the percentage of the variance of the muscle coordination patterns explained by the first 25 PCs.

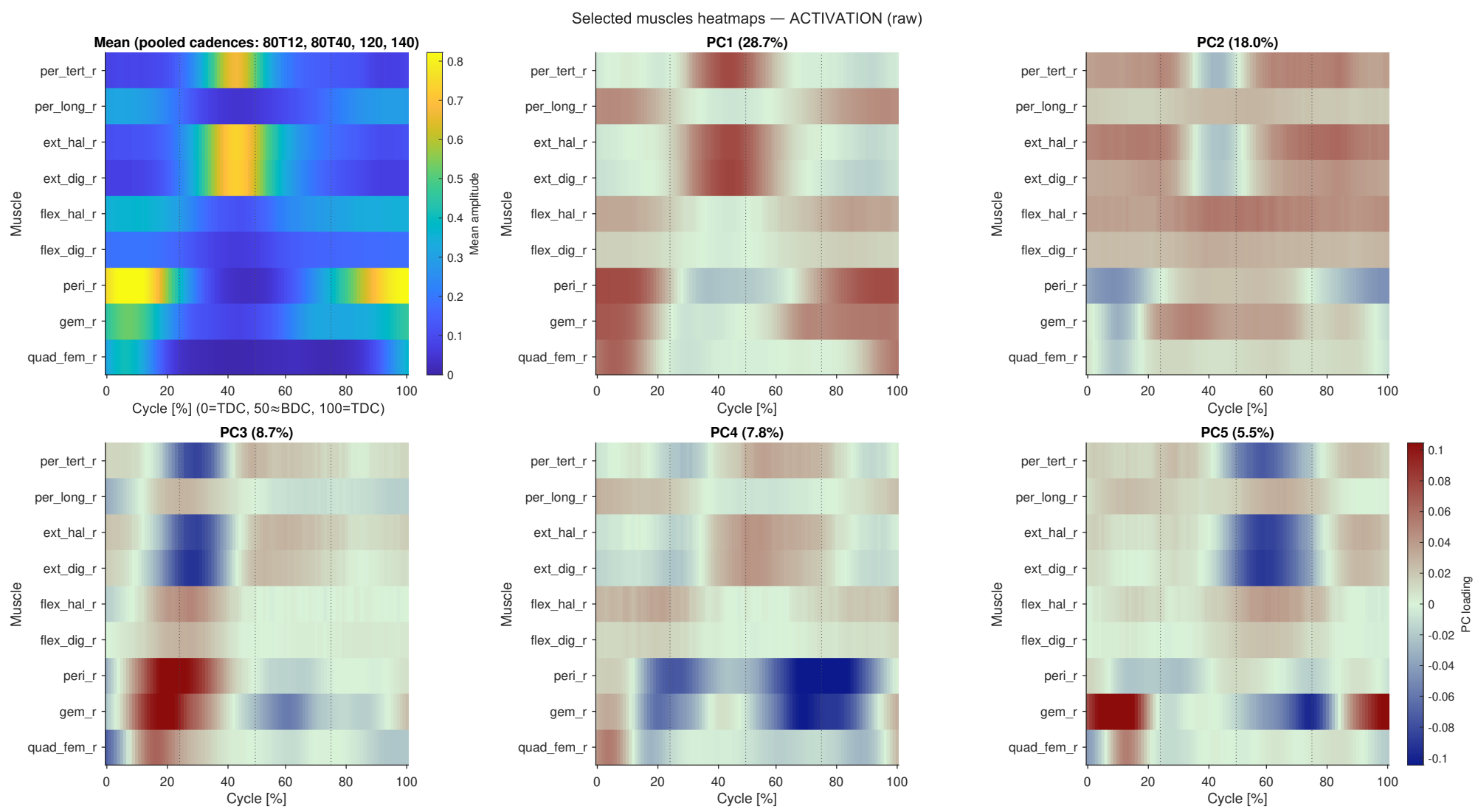

Fig. S3. The mean activation pattern and the weights for the first five Principal Components (PCs: 1-5). The variance explained is shown for each PC. Time is normalised to a percentage of the pedal cycle, starting and finishing with the crank at top-dead-centre.

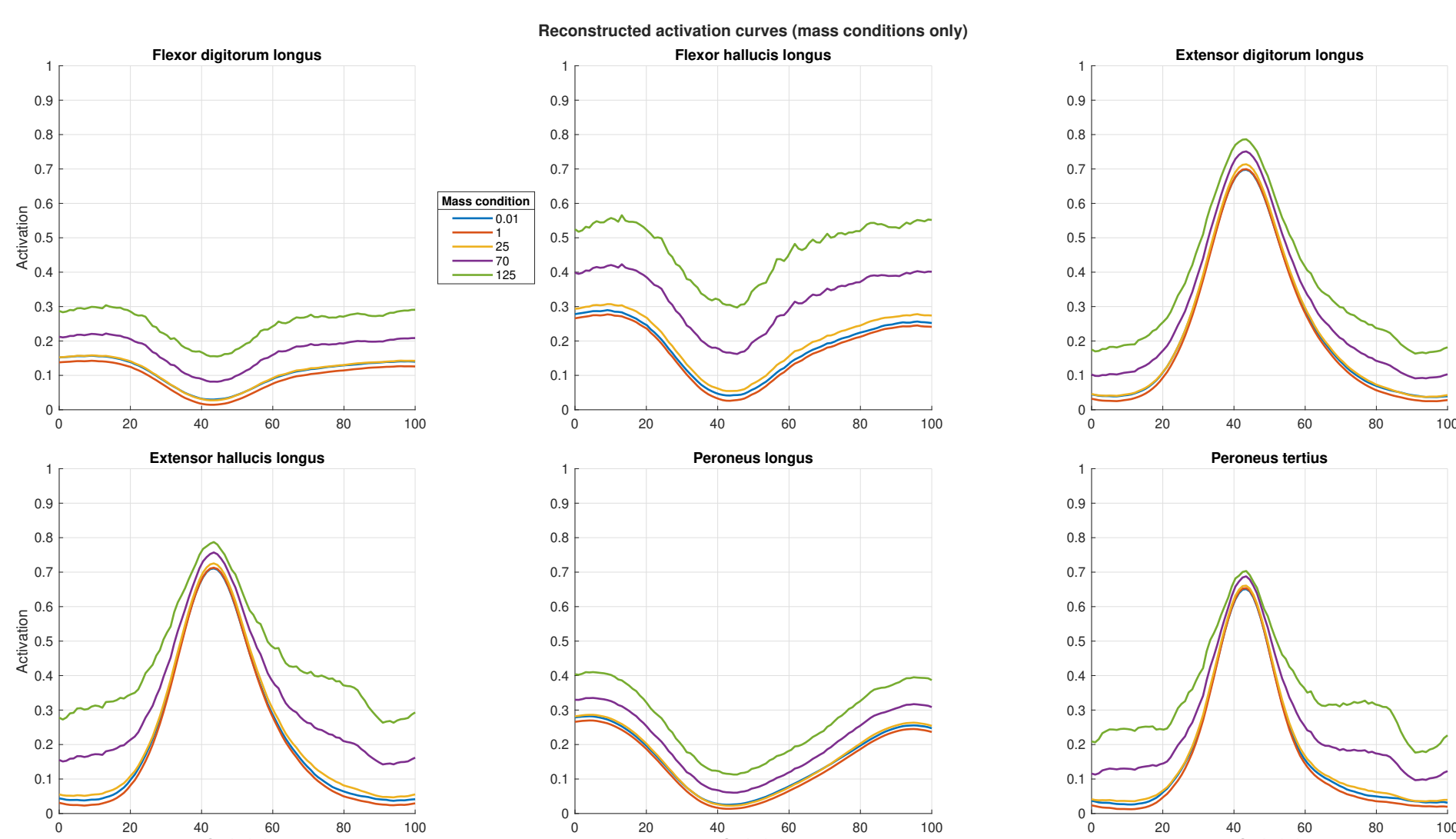

Fig. S4. Reconstructed activation patterns for the other muscles using the mean activation and vector product of the loading scores and weights for PCs1-14. The statistical effect of the muscle-mass scale is shown. Loading scores were only selected to differ between conditions if there was a significant effect of scale for that PC from the ANOVA.
